# Ruminal fermentation characteristics and related feeding values of compound feeds and their constituting single feeds studied by using *in vitro* techniques

**DOI:** 10.1017/S1751731120000889

**Published:** 2020-09

**Authors:** G. Grubješić, N. Titze, J. Krieg, M. Rodehutscord

**Affiliations:** Institut für Nutztierwissenschaften, Universität Hohenheim, Emil-Wolff-Str. 6-10, 70599 Stuttgart, Germany

**Keywords:** additivity, associative effects, *in situ* prediction, mixed feed, interaction

## Abstract

Single concentrate feeds are mixed together forming compound feeds for cattle. However, knowledge regarding the potential interactions (associative effects) between the feeding values of single feeds in compound feeds is lacking. The main objective of the present study was to evaluate ruminal fermentation characteristics and feeding values of eight industrially produced compound feeds in mash form from their constituent single feeds for dairy cows through *in vitro* assays. Additivity was given for gas production (**GP**), digestibility of organic matter (**dOM**) and utilisable CP at the duodenum (**uCP**). Additivity of CP fractions (determined using the Cornell Net Carbohydrate and Protein System (**CNCPS**)) was dependent on the fraction and compound feed type; however, the effective degradation calculated from CP fractions (**ED**_**CNCPS**_) showed additivity. Additivity was not given for intestinal digestibility of rumen-undegraded protein (**ID**_**RUP**_) for five out of eight compound feeds. Precise calculation of metabolisable energy (**ME**) of compound feeds from ME of single feeds was possible when using the same ME equations for all single and compound feeds. Compound feeds are often provided in pellet form; therefore, our second objective was to evaluate the effects of pelleting on ruminal fermentation characteristics and feeding values of compound feeds. Pelleting affected GP at 24 h (**GP**_**24**_; up to 2.4 ml/200 mg DM), dOM (up to 2.3 percentage point (**pp**)) and ME (up to 0.3 MJ/kg DM), but these differences were overall small. More considerable effects of pelleting were observed for uCP, which was increased in all compound feeds except the two with the highest CP concentrations. The ID_RUP_ was lower in most compound feeds following pelleting (up to 15 pp). Pelleting also affected CP fractions in a non-systematic way. Overall, the effects of pelleting were not considerable, which could be because pelleting conditions were mild. Our third objective was to compare *in situ* ruminal CP degradation (**ED**_**IN_SITU**_) of compound feeds with ED using two prediction methods based on CP fractions. ED_IN_SITU_ reference data were obtained from a companion study using the same feeds. Prediction accuracy of ED_IN_SITU_ and ED_CNCPS_ was variable and depended on the compound feed and prediction method. However, future studies are needed as to date not enough data are published to draw overall conclusions for the prediction of ED_IN_SITU_ from CP fractions.

## Implications

Compound feeds are often fed to high-yielding dairy cows, both in mash and pellet form. Estimation of ruminal fermentation characteristics and feeding value of compound feeds from the single feeds contained therein is necessary for efficient feeding; therefore, this was assessed in the present study. Pelleting of compound feeds had only a negligible effect on ruminal fermentation characteristics and feeding values. Predictions of ruminal protein degradation based on CP fractions of the feed were not reliable.

## Introduction

Intensive dairy cow farming is reliant on adequate feeding to satisfy the increasing nutritive requirements of cows due to increasing milk yield. Concentrate compound feeds are often included in diets of dairy cows and are either provided with forages in the form of total mixed rations or separately. The additivity of feeding values of single feeds used in compound feeds is commonly assumed based on the presumption that no interactions between single feeds exist.

*In vitro* methods are widely used for feed evaluation because *in vivo* evaluations are expensive and laborious, and they require animals (GfE, 2017). To estimate the digestibility of organic matter (**dOM**) and metabolisable energy (**ME**), measuring gas production (**GP**) by the Hohenheim gas test (**HGT**), as described by Menke and Steingass (1988), is an established assay. An extension of this method known as extended HGT (**eHGT**; Steingaß and Südekum, 2013) can be used to estimate the utilisable CP at the duodenum (**uCP**), which is the basis for the calculation of metabolisable protein used in the German protein evaluation system for cows (GfE, 2001). Calsamiglia and Stern (1995) developed a three-step method for estimating the intestinal digestibility of rumen-undegraded protein (**ID**_**RUP**_). These *in vitro* methods involve the use of ruminally fistulated animals as donors of rumen fluid. Sniffen *et al.* (1992) described a rapid CP fractionation method to be part of the Cornell Net Carbohydrate and Protein System (**CNCPS**). Therein, the CP in a feedstuff is separated into fractions by measuring N solubility. In an experiment by Chrenková *et al.* (2014), CP fractions were correlated with ruminal effective CP degradation (**ED**) values determined *in situ*. The CP fractions can be used to estimate ED values, which were found to correlate well with the ED values determined *in situ* (Shannak *et al.*, 2000). Additivity of feeding values of forages or mixes of forages and concentrates has been investigated utilising GP (Sandoval-Castro *et al.*, 2002; Robinson *et al.*, 2009; Niderkorn *et al.*, 2011) and uCP (Zhao *et al.*, 2005). However, to our knowledge, there has been no research on the additivity of ID_RUP_. Also, comprehensive data on additivity of multiple single feeds in a compound feed are not available.

Compound feeds for cattle are often in pellet form, and pelleting can increase the availability of CP and starch (**ST**) or increase indigestible bonds, depending on the intensity of the pelleting process (Svihus and Zimonja, 2011). In compound feeds, these effects can depend on the choice of single feeds, and hence, they should be examined over a wide range of various compound feeds. The objective of the present study was to characterise GP and the related values of dOM and ME as well as uCP, ID_RUP_ and CP fractions of single feeds and the compound feeds produced with them, both in mash and pellet form. Three hypotheses were developed:

(I) Values of GP, dOM, ME, uCP, ID_RUP_ and CP fractions of compound feeds in mash form can be calculated from data obtained for single feeds;
(II) Pelleting significantly affects GP, dOM, ME, uCP, ID_RUP_ and CP fractions of compound feeds;
(III) Ruminal effective degradability of CP determined *in situ* can be predicted from CP fractions.

## Material and methods

### Samples of single and compound feeds

Eight compound feeds with different target CP concentrations (16%, 18%, 20%, 22%, 24%, 26%, 28% and 30% CP in DM) were mixed using 12 single feeds: maize, wheat, barley, soya beans, soya bean meal, rapeseed meal, sunflower meal, faba beans, dried distillers’ grains with solubles (**DDGS**), maize gluten, wheat bran and sugar beet pulp. Between five and seven single feeds were included in each compound feed in different concentrations. Compound feeds were produced in mash and pellet form using standard industrial processes in the feed mill of RKW-Kehl (Kehl, Germany). Production and analysed nutrient concentrations and particle size distribution of all feeds were detailed previously (Grubješić *et al.*, 2019). Targeted CP concentrations were achieved in all compound feeds. Crude protein, ash, ether extract (**EE**), NDF assayed with a heat stable amylase and expressed exclusive of residual ash (**aNDFom**) and ADF expressed exclusive of residual ash (**ADFom**) did not differ more than one percentage point (**pp**) between calculated concentrations from single feeds and analysed concentrations in mash compound feeds.

### Gas production kinetics, metabolisable energy and digestibility of organic matter

*In vitro* GP kinetics were measured using HGT following the procedure described by Seifried *et al.* (2016). Approximately 200 ± 5 mg of feed ground through a 1-mm sieve was transferred into graded glass syringes (100 ml volume). Fresh rumen fluid was obtained from two rumen-fistulated Jersey cows, one lactating and one not lactating. The lactating cow was provided a ration consisting of (on DM basis) 41.3% concentrate mix, 20.0% maize silage, 16.3% meadow hay, 15.0% grass silage, 3.6% barley straw, 2.4% mineral mix and 1.4% rapeseed meal. The other cow was provided a ration consisting of 35.4% maize silage, 35.4% grass silage, 24.6% meadow hay, 3.2% barley straw, 1.0% mineral mix and 0.4% urea. Cows had *ad libitum* access to feed.

The rumen fluid obtained from the two cows was mixed to a 1 : 1 ratio, filtered through two layers of cheesecloth, and a reduced buffer solution was added. Syringes were pre-warmed to 39°C before 30 ml of buffer-rumen fluid mix was poured into each syringe under constant CO_2_ flow. Each feed was included in five separate HGT runs with two replicated syringes per feed in each run. Additionally, each run contained three syringes without feed samples (blanks) and three syringes with a concentrate standard feed. Cumulative GP was recorded after 2, 4, 6, 8, 12, 24, 48 and 72 h of incubation at 39°C under constant rotation. The following non-linear regression was fitted to the obtained GP data according to Seifried *et al.* (2016):(1)
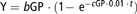
where *b*GP is the potential GP (ml/200 mg DM), *c*GP the rate of GP (%/h) and *t* the incubation time (h).

The dOM was calculated using GP at 24 h (**GP**_**24**_) corrected for the blanks and standard (GP_24_; ml/200 mg DM) and chemical analysis according to Menke and Steingass (1988):(2)
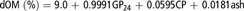



The ME was calculated using GP_24_ and specific to the type of feed, as follows:

(a) Maize, wheat, barley, faba beans, maize gluten and wheat bran according to Krieg *et al.* (2017):(3)
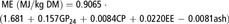

(b) Non-cereal feeds (soya beans, soya bean meal, rapeseed meal, sunflower meal, DDGS and sugar beet pulp) according to Menke and Steingass (1988):(4)
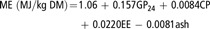

(c) Compound feeds according to GfE (2009):(5)
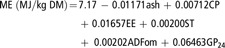

In equations (2) to (5), CP, ST, EE, ash and ADFom are expressed in g/kg DM.

### Utilisable CP at the duodenum

The eHGT method described by Steingaß and Südekum (2013) was used to estimate uCP and was conducted according to Westreicher-Kristen *et al.* (2015). Some former studies using this approach used the term ‘modified HGT’ and the abbreviation ‘modHGT’. However, the term ‘extended HGT’ and the abbreviation ‘eHGT’ may be more appropriate as this method is not a real modification of the original HGT but an extension (measuring NH_3_-N after incubation) and can be connected with GP_24_ measurement to estimate dOM and ME. Samples were incubated similarly to those in the HGT method described above. Donor cows had *ad libitum* access to a ration consisting of (on DM basis) 25.8% concentrate mix, 24.3% grass silage, 24.3% maize silage, 17.0% hay, 4.4% rapeseed meal, 2.2% barley straw and 2.0% mineral mix. Samples were incubated twice for different times (8 and 24 h), and a standard concentrate sample with known uCP concentration was included to check the variation of uCP results among runs. Each feed sample was incubated in five separate runs per incubation time. Following incubation, all syringes were rapidly frozen to minimise microbial fermentation. The following day, the NH_3_-N concentration of incubation residues obtained from the syringes was analysed (Vapodest 50; C. Gerhardt GmbH & Co. KG, Königswinter, Germany*).* The NH_3_-N concentration was used to estimate the uCP concentration as follows:(6)
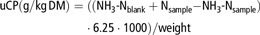
where N_sample_ is the amount of N from the feed sample (mg), NH_3_-N_sample_ and NH_3_-N_blank_ are the NH_3_-N content of feed samples and blank incubation residues (mg) and weight is the weight of feed sample inserted into the glass syringe (mg DM). Effective uCP was estimated for theoretical ruminal passage rates (*k*) of 5 and 8%/h by plotting uCP values (y) against the natural logarithm of the incubation time (x) in a linear regression model and calculating the function values of ln (20) and ln (12.5), respectively (Steingaß and Südekum, 2013).

### Intestinal digestibility of rumen-undegraded protein

The three-step enzymatic method of Calsamiglia and Stern (1995) was used to determine ID_RUP_. Samples of single and compound feeds were ground through a 2-mm screen. The first step was a 16-h *in situ* incubation in the rumen, and this was conducted using three rumen-fistulated Jersey cows, following the procedure described in Seifried *et al.* (2016). A minimum of 60 mg of residual N per feed was accumulated for subsequent *in vitro* simulation of digestibility in the abomasum and duodenum. Two or three samples per feed containing 15 mg of residual N were incubated utilising 10 ml HCl (0.1 N, pH = 1.9), pepsin (1 g/l, Sigma P-7012; Sigma, St. Louis, MO, USA) and pancreatin solution (0.5 M KH_2_PO_4_ buffer standardised at pH 7.8 containing 50 ppm of thymol and 3 g/l of pancreatin, Sigma P-7545; Sigma). Trichloroacetic acid was added to stop enzymatic action and precipitate undigested proteins. Samples were centrifuged at 15 000 g for 25 min. Supernatants were analysed for soluble N by the Kjeldahl method (VDLUFA, 2007). Finally, ID_RUP_ was calculated as follows:(7)
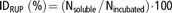
where N_soluble_ is the amount of soluble N determined *in vitro* (mg) and N_incubated_ is the total N that was incubated with pepsin and pancreatin (mg).

### CP fractionation

Crude protein fractions were estimated according to the CNCPS (Sniffen *et al.*, 1992): fraction A represented the non-protein N, fraction B the true protein and containing three sub-fractions (B1 to B3) differing in their rate of ruminal degradation and fraction C the acid detergent insoluble N. To calculate CP fractions, non-protein N, buffer-soluble protein, neutral detergent insoluble N and acid detergent insoluble N were determined according to Licitra *et al.* (1996) for all samples of single and compound feeds. Table values of ruminal degradation rates of CP fractions of single feeds (Fox *et al.*, 2003) were used together with determined CP fractions to calculate ED_CNCPS_ using equation (8) (Fox *et al.*, 2003):(8)
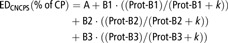



where ED_CNCPS_ is ED calculated from CP fractions, A and B1 to B3 are determined CP fractions, Prot-B1 to Prot-B3 are table values for ruminal degradation rates of CP fractions and *k* is the ruminal passage rate (5 or 8%/h). The ruminal degradation rate of faba beans was not reported in Fox *et al.* (2003), and thus, the value for lupins was used instead as they both belong to the legume family and show similar ED values (Goelema *et al*, 1998). Table values of ruminal degradation rates of compound feeds were not available; therefore, they were calculated from values of the respective single feeds. This calculation included weighting contributions of CP of single feeds to the total CP of the respective compound feed. Ruminal degradation rates were then used together with determined CP fractions to estimate the observed ED_CNCPS_ for mash and pelleted compound feeds.

An alternative prediction equation (Shannak *et al.*, 2000) based on CP fractions and NDF was used to estimate RUP of compound feed as follows:(9)
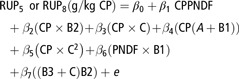
where RUP_5_ or RUP_8_ are RUP values for rumen outflow rates of *k* = 5 and 8%/h, respectively. The CPPNDF is the CP concentration in PNDF (NDF determined by manual filtration on paper) and all nutrients are given as g/kg DM, whereas A, B and C fractions are given as g/kg CP. Instead of CPPNDF and PNDF, the CP concentration in aNDF as well as aNDF was determined in the present study by the conventional method (VDLUFA, 2007). The general form of the equation is identical for RUP_5_ and RUP_8_, and the parameter estimated of β_0_ to β_7_ is given in Shannak *et al.* (2000). The ED_CNCPS_ was then calculated from RUP values for the given rumen outflow as:(10)
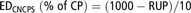



The ED_CNCPS_ values of compound feeds (calculated either with equation (8) or with equations (9) and (10)) were compared with measured ED_IN_SITU_ values of a companion study that determined *in situ* degradation values of the same feeds used in the present study (Grubješić *et al.*, 2019).

### Additivity calculation

To evaluate the additivity of all traits of single feeds in a mash compound feed, the expected value of the compound feed was calculated based on weighted contribution of DM (for *b*GP, *c*GP, GP_24_, dOM, ME and uCP) or CP (for ID_RUP_ and CP fractions) from single feeds to the DM and CP contained in the respective compound feed. These values are referred to as ‘calculated’ herein. To calculate the ME values of compound feeds from single feeds, two approaches were used. The ME values of single feeds were determined according to either equations (3) or (4) depending on the feed group or alternatively equation (5) for all single feeds.

### Statistical analyses

Calculated and observed values of mash compound feeds, and values of mash and pellet compound feeds, were regressed using procedure REG (version 9.4 of SAS system for Windows SAS Institute, Cary, NC, USA). The REG procedure was also used to calculate if slopes and intercepts were significantly different from 1 and 0, respectively, by determination of 95% CI to detect possible associative effects.

## Results

### *In vitro* ruminal fermentation and feeding values of single feeds

Overall, ruminal fermentation characteristics and feeding values of single feeds varied widely (Table 1). The highest GP_24_ was found in maize (81 ml/200 mg DM) and the lowest in sunflower meal (36 ml/200 mg DM). The highest dOM was found in maize and wheat (96%) and the lowest in sunflower meal (65%). The highest ME was found in soya beans (16.0 MJ/kg DM) followed by maize (14.5 MJ/kg DM) and wheat (14.2 MJ/kg DM) and the lowest in sunflower meal (9.4 MJ/kg DM). The uCP concentration varied between 144 g/kg DM in wheat bran and 279 g/kg DM in soya bean meal for *k* = 5%/h, and between 158 g/kg DM in maize and wheat bran and 356 g/kg DM in soya bean meal for *k* = 8%/h. The ID_RUP_ ranged between 18% in wheat bran and 83% in maize and soya bean meal. All CP fractions varied widely between single feeds. This was reflected in the high variability of ED_CNCPS_ values.

**Table 1.** In vitro *fermentation characteristics and feeding value of single feeds*

Single feed	*In vitro* gas production			dOM	ME	uCP		ID_RUP_	CP fractions					ED_CNCPS_	
	*b*GP (ml/200 mg DM)	*c*GP (%/h)	GP_24_ (ml/200 mg DM)	(%)	(MJ/kg DM)	*k* = 5%/h (g/kg DM)	*k* = 8%/h (g/kg DM)	(%)	A (%)	B1 (%)	B2 (%)	B3 (%)	C (%)	*k* = 5%/h (%)	*k* = 8%/h (%)
Maize	92	5.7	81	96	14.5	163	158	83	12.5	5.9	66.6	14.9	0.0	65	55
Wheat	70	8.3	78	96	14.2	180	198	35	9.8	22.2	63.5	4.4	0.0	80	73
Barley	81	7.7	75	92	13.6	162	175	47	11.4	16.4	62.3	5.0	5.0	76	69
Soya beans	47	10.8	46	79	16.0	227	282	71	4.5	4.8	82.7	6.4	1.6	56	45
Soya bean meal	56	8.0	51	92	13.3	279	356	83	2.9	8.4	83.8	3.6	1.2	72	62
Rapeseed meal	50	8.5	45	78	11.3	241	280	26	7.1	13.4	62.5	10.2	6.8	73	65
Sunflower meal	39	8.9	36	65	9.4	166	205	46	8.3	27.3	54.6	5.9	3.9	76	69
Faba beans	70	8.3	63	90	12.9	202	224	32	12.7	42.3	38.7	4.2	2.1	80	75
DDGS	51	8.7	44	73	11.9	275	296	75	20.6	0.0	44.1	11.8	23.6	55	50
Maize gluten	62	6.8	54	78	11.0	188	196	59	48.5	6.2	33.8	8.6	2.9	79	75
Wheat bran	57	9.9	52	72	10.9	144	158	18	12.5	21.1	49.4	13.6	3.4	80	74
Sugar beet pulp	78	9.4	74	90	13.2	182	199	67	39.9	0.0	29.2	24.8	6.2	82	78

*b*GP = potential gas production; *c*GP = rate of gas production; GP_24_ = corrected gas production at 24 h; dOM = digestibility of organic matter; ME = metabolisable energy; uCP = utilisable CP for ruminal passage rates (*k*) of 5 and 8%/h; ID_RUP_ = intestinal digestibility of rumen-undegraded protein; CP fractions = crude protein fractions: A = non-protein nitrogen; B1 = rapidly degradable true protein; B2 = moderately degradable true protein; B3 = slowly degradable true protein, C = undegradable and indigestible true protein, determined using Cornell Net Carbohydrate and Protein System (CNCPS); ED_CNCPS_ = effective protein degradation for ruminal passage rates of 5 and 8%/h, calculated using Fox *et al.* (2003); DDGS = dried distillers’ grains with solubles.

### Additivity of fermentation characteristics and feeding values

**Figure 1.**
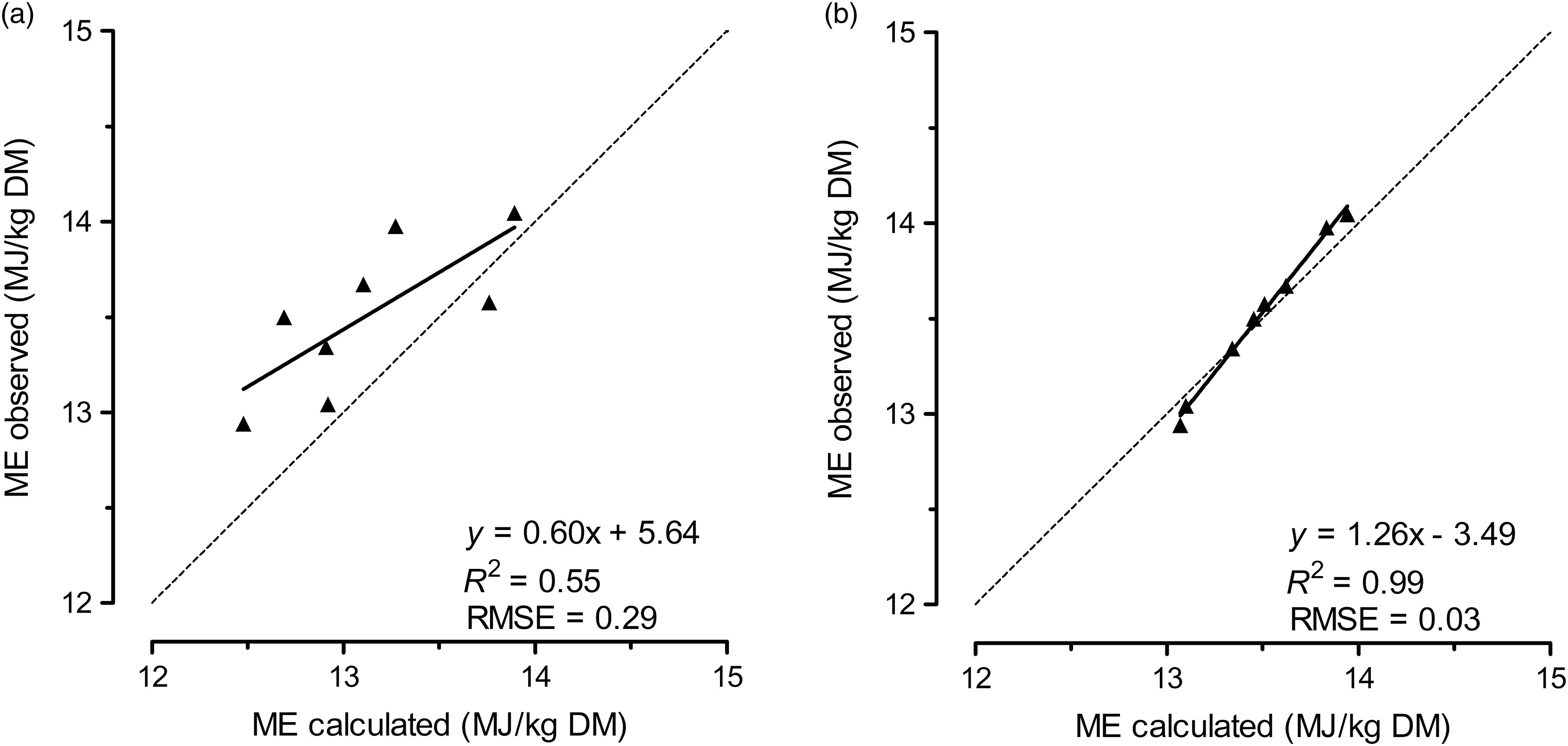
Comparison of calculated and observed metabolisable energy (ME) values of compound feeds using an *in vitro* ruminal fermentation technique. The ME values of compound feeds were calculated from ME values of single feeds that were determined according to the equations of: (a) Krieg *et al.* (2017) and Menke and Steingass (1988), respective of the feed group; or (b) GfE (2009) for all single feeds. The dotted line represents the angle bisector.

Calculated and observed ruminal fermentation characteristics and nutritional values of the mash compound feeds are presented in Table 2. Estimation of *b*GP, GP_24_ and dOM was precise as indicated by the slope of the regression lines (close to 1) and the high *R* ^2^ values (Table 3). Observed *c*GP differed numerically (0.3 to 0.7 pp) from calculated values, and the estimated slope of regression was only 0.68, associated with a large CI. Deviation of calculated and observed ME values was high when the specific equations for each group of feed were used. The comparison between calculated and observed ME showed a low *R* ^2^ value of 0.55 with a RMSE of 0.29 (Figure 1). However, when using the same ME equation (equation (5)) for all single and compound feeds, estimated ME of compound feeds from that of single feeds was precise, with an *R* ^2^ value of 0.99 (Figure 1).

**Table 2.** In vitro *fermentation characteristics and feeding value of mash and pellet compound feeds and values calculated from single feeds*

		*In vitro* gas production			dOM	uCP		ID_RUP_	CP fractions					ED_CNCPS_	
Compound feed		*b*GP (ml/200 mg DM)	*c*GP (%/h)	GP_24_ (ml/200 mg DM)	(%)	*k* = 5%/h (g/kg DM)	*k* = 8%/h (g/kg DM)	(%)	A (%)	B1 (%)	B2 (%)	B3 (%)	C (%)	*k* = 5%/h (%)	*k* = 8%/h (%)
1	Calculated	77	7.0	73	92	181	193	63	12.9	12	66.3	7.9	0.9	75	67
	Mash	81	7.3	74	93	173	188	71	17.1	7.1	68.0	7.8	0.0	75	67
	Pellet	77	8.4	71	91	192	212	73	19.4	3.0	69.9	7.7	0.0	74	66
2	Calculated	69	7.9	66	87	190	205	46	16.5	11.8	54.5	10.8	6.4	76	69
	Mash	71	8.6	65	86	177	195	55	23.2	3.9	55.3	14.1	3.5	76	69
	Pellet	70	9.2	65	86	185	204	48	21.7	5.9	54.9	14.0	3.5	76	69
3	Calculated	68	8.2	62	84	175	193	44	17.3	21.3	51.3	6.8	3.2	77	70
	Mash	67	8.3	61	83	173	188	49	18.1	19.5	52.9	6.3	3.2	77	70
	Pellet	67	9.1	62	83	178	198	34	17.4	16.9	56.3	6.3	3.2	76	69
4	Calculated	72	8.2	66	89	192	216	59	9.3	15.3	65.3	6.0	4.1	71	62
	Mash	73	7.5	67	90	186	210	70	8.8	14.7	67.9	5.7	2.9	71	62
	Pellet	72	8.5	66	89	190	214	63	10.1	15.6	65.5	5.8	2.9	72	63
5	Calculated	68	7.6	63	87	200	222	49	9.3	18.6	59.8	7.7	4.6	73	66
	Mash	69	8.1	62	86	191	209	58	10.1	18.9	57.8	7.9	5.3	73	66
	Pellet	68	9.0	63	86	190	213	51	9.0	17.8	62.2	5.5	5.5	72	65
6	Calculated	63	8.7	58	83	196	225	49	9.3	19.0	61.1	7.3	3.3	73	66
	Mash	64	8.4	59	84	189	213	49	9.0	21.1	60.0	7.4	2.5	74	66
	Pellet	66	8.9	61	85	194	217	44	9.5	19.9	59.9	5.4	5.4	71	64
7	Calculated	55	8.9	54	81	199	233	50	9.3	14.1	66.4	7.1	3.0	73	65
	Mash	59	9.2	54	81	195	226	53	9.4	14.4	64.9	9.0	2.2	73	65
	Pellet	59	9.5	55	81	188	220	41	10.5	12.6	67.5	7.1	2.4	72	64
8	Calculated	62	8.7	57	85	203	240	60	7.1	17.9	67.6	5.0	2.4	73	65
	Mash	64	8.4	59	87	199	237	56	9.4	18.4	66.0	4.2	2.1	73	65
	Pellet	63	9.2	59	86	198	233	48	7.7	17.8	68.0	4.3	2.1	72	64

*b*GP = potential gas production; *c*GP = rate of gas production; GP_24_ = corrected gas production at 24 h; dOM = digestibility of organic matter; uCP = utilisable CP for ruminal passage rates (*k*) of 5 and 8%/h; ID_RUP_ = intestinal digestibility of rumen-undegraded protein; CP fractions = crude protein fractions: A = non-protein nitrogen; B1 = rapidly degradable true protein; B2 = moderately degradable true protein; B3 = slowly degradable true protein, C = undegradable and indigestible true protein, determined using Cornell Net Carbohydrate and Protein System (CNCPS); ED_CNCPS_ = Effective protein degradation for ruminal passage rates of 5 and 8%/h, calculated using Fox *et al.* (2003). Metabolisable energy (ME) values are presented in Figure 1.

**Table 3.** *Results of simple linear regressions for* in vitro *fermentation characteristics and feeding values of compound feeds*

	Calculated *v*. observed						Mash *v*. pelleted					
	Slope	Slope CI	Intercept	Intercept CI	*R* ^2^	RMSE	Slope	Slope CI	Intercept	Intercept CI	*R* ^2^	RMSE
*In vitro* gas production												
* bGP*	0.98	0.74 to 1.21	3.58	−11.95 to 19.11	0.95	1.70	0.79	0.64 to 0.93	13.80	3.87 to 23.72	0.97	1.06
* c*GP	0.68	0.03 to 1.33	2.67	−2.65 to 7.98	0.52	0.44	0.59	0.43 to 0.75	4.14	2.82 to 5.47	0.93	0.10
* *GP_24_	0.96	0.78 to 1.14	2.72	−8.52 to 13.95	0.97	1.19	0.82	0.67 to 0.98	11.27	1.63 to 20.91	0.97	1.00
dOM	1.10	0.73 to 1.40	−4.95	−33.43 to 23.53	0.91	1.25	0.77	0.59 to 0.94	19.84	4.66 to 35.02	0.95	0.74
ME	–^1^	–	–	–	–	–	0.71	0.54 to 0.87	3.82	1.61 to 6.03	0.95	0.07
uCP												
* k* = 5%/h	0.97	0.61 to 1.33	−1.05	−70.00 to 67.89	0.88	3.73	0.37	−0.11 to 0.85	120.09	31.12 to 209.07	0.38	5.16
* k* = 8%/h	0.97	0.77 to 1.17	−1.26	−45.18 to 42.66	0.96	3.89	0.53	0.29 to 0.77	103.00	53.24 to 152.76	0.83	4.53
ID_RUP_	0.95	0.23 to 1.66	7.86	−30.08 to 45.80	0.64	5.53	1.42	0.99 to 1.85	−31.27	−56.26 to −6.28	0.92	3.94
CP fractions												
* *A	1.37	0.79 to 1.94	−24.19	−92.26 to 43.88	0.85	23.09	0.95	0.69 to 1.20	7.15	−28.95 to 43.25	0.93	15.30
* *B1	1.65	0.94 to 2.35	−119.87	−237.17 to −2.56	0.84	26.55	0.94	0.64 to 1.24	−2.20	−49.96 to 45.55	0.91	20.23
* *B2	0.94	0.64 to 1.23	38.34	−144.37 to 221.05	0.91	19.22	0.88	0.52 to 1.24	88.96	−131.32 to 309.24	0.86	22.79
* *B3	1.70	1.22 to 2.18	−46.79	−82.89 to −10.69	0.93	8.71	0.94	0.57 to 1.32	−3.55	−34.68 to 27.58	0.86	12.03
* *C	0.71	0.13 to 1.29	2.36	−19.67 to 24.40	0.60	10.21	0.98	0.31 to 1.65	4.60	−15.84 to 25.04	0.68	10.83
ED_CNCPS_												
* k* = 5%/h	1.24	−0.41 to 2.89	−12.35	−134.36 to 109.66	0.36	3.40	0.63	0.14 to 1.11	31.83	−7.02 to 70.68	0.62	2.08
* k* = 8%/h	1.41	−0.20 to 3.02	−20.20	−126.68 to 86.28	0.43	4.22	0.65	0.19 to 1.10	28.54	−4.71 to 61.79	0.67	2.55

*b*GP = potential gas production; *c*GP = rate of gas production; GP_24_ = corrected gas production at 24 h; dOM = digestibility of organic matter; ME = metabolisable energy; uCP = utilisable CP for ruminal passage rates (*k*) of 5 and 8%/h; ID_RUP_ = intestinal digestibility of rumen-undegraded protein; CP fractions = crude protein fractions: A = non-protein nitrogen; B1 = rapidly degradable true protein; B2 = moderately degradable true protein; B3 = slowly degradable true protein, C = undegradable and indigestible true protein, determined using Cornell Net Carbohydrate and Protein System (CNCPS); ED_CNCPS_ = effective protein degradation for ruminal passage rates of 5 and 8%/h, calculated using Fox *et al.* (2003). Observed values refer to values for compound feeds in mash form.

1 Simple linear regressions of calculated and observed ME values are presented in Figure 1.

Observed uCP was numerically lower than calculated in all compound feeds. However, the difference did not exceed 13 g/kg DM. The regression line slopes were close to 1 (0.97 for both *k* = 5 and 8%/h) and regression equations showed high *R* ^2^ values (0.88 and 0.96 for *k* = 5 and 8%/h, respectively).

Observed ID_RUP_ in compound feeds differed from calculated values between 0 and 11 pp. Regression analysis between calculated and observed ID_RUP_ values showed an *R*² value of 0.64 and relatively large CIs for the slope and intercept, respectively.

Conformity between calculated and observed CP fractions depended on the specific fraction and the compound feed type. Confidence interval of the slope did not include the value of 1 only for B3 (CI = 1.22 to 2.18), even though the *R* ^2^ value for this parameter was high (*R* ^2^ = 0.93).

Results of the regression analysis of calculated and observed ED_CNCPS_ values showed small accuracy (*R* ^2^ of 0.36 and 0.43 for *k* = 5 and 8%/h, respectively). However, the slope values included 1 and intercept values included 0 and numerical differences were in most cases not even detectable (Table 2).

### Effects of pelleting on ruminal fermentation characteristics and feeding value of compound feeds

Differences between mash and pellet compound feeds in GP_24_ did not exceed 3 ml/200 mg DM, 3 pp in dOM and 0.3 MJ ME/kg DM (Table 2). However, based on CI ranges (Table 3), the results indicated that pelleting did affect GP characteristics. The slopes and the intercepts for *b*GP, *c*GP, GP_24_, dOM and ME were all significantly different from 1 to 0, respectively, even though the *R* ^2^ value was 0.93 or higher. Pelleting numerically increased uCP in compound feeds with lower CP concentration and decreased uCP in compound feeds with higher CP concentration. The slopes and intercepts were significantly different from 1 and 0, respectively, with considerable differences in *R* ^2^ values between *k* = 5%/h (*R* ^2^ = 0.38) and *k* = 8%/h (*R* ^2^ = 0.83). Pelleting decreased estimated ID_RUP_ in most compound feeds, with a maximum of 15 pp in compound feed 3. Pelleting increased estimated ID_RUP_ only in compound feed 1, but the difference was negligible (2 pp). Although the CI for the slope of ID_RUP_ included 1 and the *R* ^2^ value was high (*R* ^2^ = 0.92), the intercept was significantly different from 0. Pelleting did not systematically affect CP fractions in compound feeds (Table 3). Pelleting reduced the ED_CNCPS_ in most compound feeds slightly (up to 3 pp) for both *k* = 5 and 8%/h.

### Prediction of *in situ* ruminal CP degradation from CP fractions

**Figure 2.**
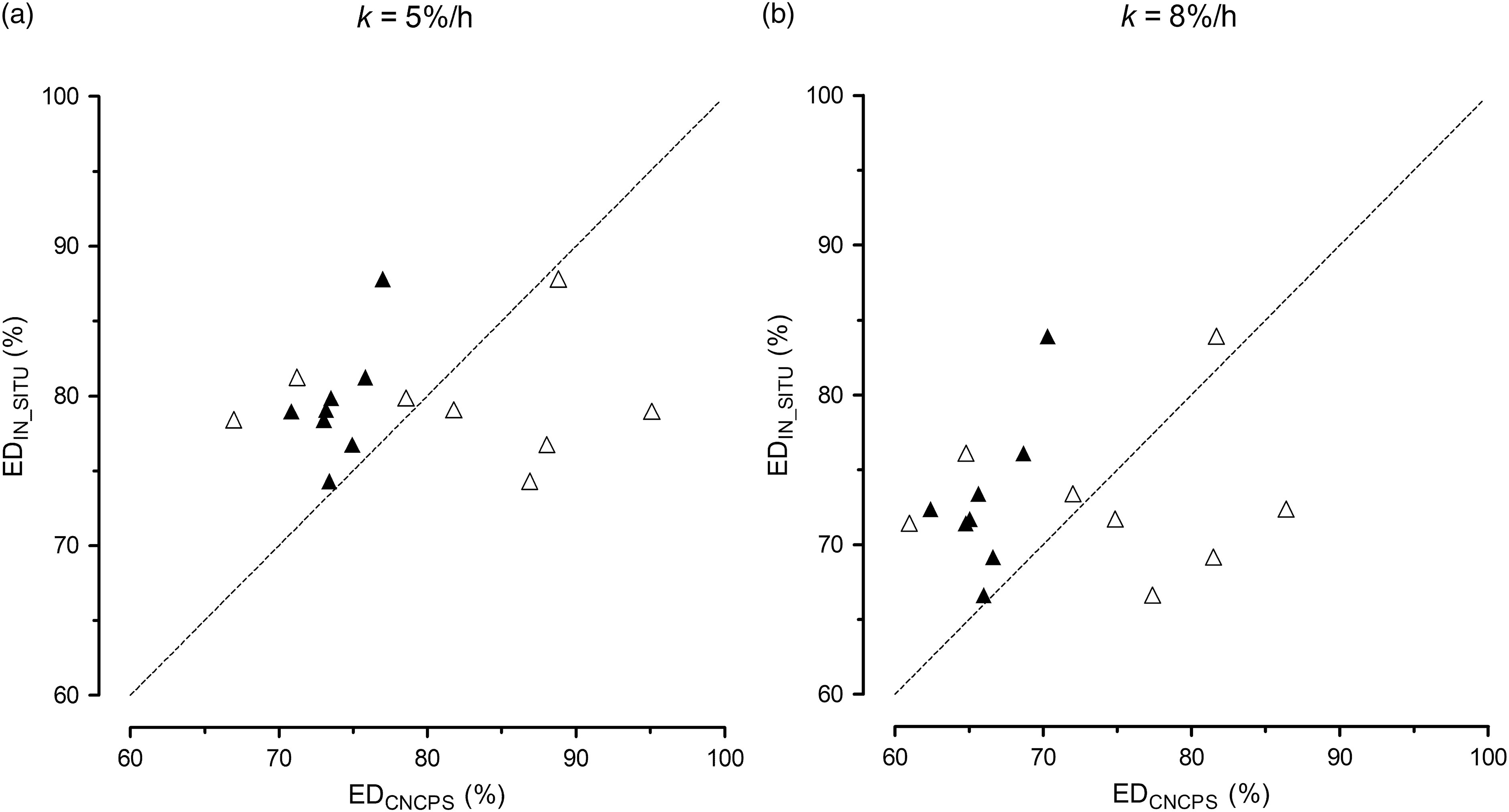
Comparison of ruminal effective protein degradation of compound feeds for ruminal passage rates of 5 and 8%/h based on CP fractions (ED_CNCPS_) and calculated according to Fox *et al.* (2003) (▴) or Shannak *et al.* (2000) (▵) and determined *in situ* (ED_IN_SITU_; Grubješić *et al.*, 2019). The dotted line represents the angle bisector.

The ED_CNCPS_ values were smaller than ED_IN_SITU_ for all compound feeds, and the difference was up to 11 and 14 pp for *k* = 5 and 8%/h, respectively (Figure 2). Calculation of ED_CNCPS_ using individual CP fractions and tabular values for their specific degradation rates resulted in a very low variation from 71% to 77% (*k* = 5%/h) and 62% to 70% (*k* = 8%/h), whereas ED_IN_SITU_ of compound feeds showed wider variation from 74% to 88% (*k* = 5%/h) and 67% to 84% (*k* = 8%/h). The ED_CNCPS_ based on the regression analysis according to Shannak *et al.* (2000) resulted in a remarkable higher variability between compound feeds (from 67% to 95% for *k* = 5%/h and 61% to 86% for *k* = 8%/h) compared to ED_IN_SITU_ and ranked feeds differently (Figure 2).

## Discussion

### Additivity of ruminal fermentation characteristics and feeding values

It was hypothesised that values of GP, dOM, ME, uCP, ID_RUP_ and CP fractions of compound feeds in mash form can be calculated from single feeds. Based on the results of the present study, this hypothesis can be accepted only in part. The agreement between calculated and observed values was good for *b*GP, *c*GP, GP_24_, dOM and uCP, and thus, we consider these criteria to be additive. The highest deviation of calculated from observed value was 4 ml/200 mg DM for *b*GP, 0.7%/h for *c*GP, 2.3 ml/200 mg DM for GP_24_ and 2 pp for dOM.

For ME calculations, the equations chosen for single feeds largely affected the outcome of the comparison (Figure 1). In the literature, equations to predict ME from GP and nutrient concentrations are often specific to single feeds or groups of feeds because this is associated with high prediction accuracy. However, it does not necessarily hold true across different feed groups and also for feed mixtures (Menke and Steingass, 1988). This caused issues when values for single feeds and related compound feeds were compared in the present study. We argue that the conclusion that additivity does not exist is misleading because it is an artefact of using different equations for different groups of feeds. When equation (5) was used to calculate the ME for all feeds (single and compound), the differences between calculated and observed ME became negligible (Figure 1).

Gas production techniques are underutilised for estimation of potential interactions between feeds (D’Mello, 2000) and energy evaluation, and previous research on additivity has most commonly focused on concentrate–forage mixes. Robinson *et al.* (2009) noted associative effects (15% to 25%) in mixture of alfalfa hay, barley grain, maize silage and soya bean meal using HGT in early phase of incubation, while they disappeared later. Similarly, Arhab *et al.* (2010) used mixtures of triticale and barley with a commercial concentrate supplement and found significant differences only in GP up to 8 h. In the present study, the discrepancy between calculated and observed GP at multiple time points can be considered to be negligible with a maximal difference of 2 ml/200 mg DM after 2 and 4 h of incubation. The observed GP_24_ was similar to the calculated ones and was considered to be additive. Since dOM and ME values are derived from GP_24_ (Menke and Steingass, 1988), this is an important finding and we presume that the differences between calculated and observed ME of compound feeds were caused only by the choice of ME equation. For the estimation of dOM values, the SD of regression residuals (*s*_*y.x*_) is 3.07% (Menke and Steingass, 1988). The *s*_*y.x*_ for estimation of ME values depends on the regression equation used, with a *s*_*y.x*_ of 2.92 MJ/kg DM (Menke and Steingass, 1988) or a RMSE value of 1.98 MJ/kg DM (GfE, 2009) for equations (4) and (5), respectively. The RMSE between calculated and observed dOM and ME (equation (5)) of the compound feeds of the present study was 1.15% and 0.09 MJ/kg DM, respectively, and therefore markedly lower compared to the *s*_*y.x*_ and the RMSE of the prediction equations. This underlines the assumption that additivity of those values is given.

The uCP consists of the RUP and microbial CP, as defined in the German feed protein evaluation system (GfE, 2001). Calculated uCP values corresponded well with observed values as the slope of regression and intercept was within their CI. A systematic overestimation of uCP was observed (up to 13 g/kg DM). Repeated measurements of observed uCP for each feed and incubation time were close together and showed low SD between runs up to 23 g/kg DM. However, also variation of observed and calculated uCP between compound feeds was low for both rumen outflow rates with a maximal differences of 49 g/kg DM. Due to the small variability between feeds and only eight data points for regression analysis, this systematical overestimation should be interpreted with caution and reference to their biological and practical relevance, which seems negligible. This contrasts with the findings of Zhao *et al.* (2005), who reported higher differences between calculated and observed uCP values in an experiment using 16 single feeds and 19 mixtures. They noted statistically significant and non-systematic differences between calculated and observed uCP values. However, the authors mentioned the possibility of incomplete incubation of some feeds due to incomplete mixing with the incubation liquid (Zhao *et al.*, 2005). Such an effect was avoided in the present study by the constant motion of the rotary incubator.

Calsamiglia and Stern (1995) highlighted the importance of ID_RUP_ for evaluating feed protein. GfE (2001) and NRC (2001) assumed a constant ID_RUP_ value of 80% for all feeds. In the CNCPS, ID_RUP_ was assumed to be 100% for CP fractions A, B1 and B2, 80% for fraction B3 and zero for fraction C (Fox *et al.*, 2003). A wide range of ID_RUP_ of single and compound feeds was found in the present study. Observed ID_RUP_ in most compound feeds was higher than calculated (up to 11 pp). Accurate estimation of ID_RUP_ from single feeds was thus not possible for all compound feeds of the present study using the three-step method. This is underlined by the analytical tolerance of the determination of ID_RUP_ which was set to maximal 10% relative deviation from the mean value otherwise the procedure was repeated. Relative deviation of replicates varied between 0.04 and 8.11% around the mean value for all feed samples of the present study. However, for five out of eight compound feeds, relative deviations of calculated ID_RUP_ from observed ID_RUP_ exceeded the value of 10%, which represents the analytical tolerance. This indicates that associative effects occurred when analysing ID_RUP_ of compound feeds. Those interactions between single feeds in mixture can occur in any of the three steps. Calculation of the 16 h *in situ* CP degradation of compound feeds from single feeds showed better additivity than ID_RUP_ with a slight tendency to overestimate CP degradation (1 to 7 pp). Compound feeds that showed higher deviations between calculated and observed *in situ* degradation also tended to show higher differences between the calculated and observed ID_RUP_. Hence, associative effects seem more pronounced during the *in vitro* enzymatic part but play also a role in the first step of *in situ* incubation. As it seems that majority of the associative effects in the three-step method occurred during the *in vitro* enzymatic part, the results of the present study should be verified using the mobile bag technique as an alternative to the second step.

To our knowledge, CP fractions have not been previously studied for additivity. In the present study, observed CP fractions of mash compound feeds were often different from those calculated, as indicated by intercept values (for fractions B1 and B3 CI not including 0) and slopes (fraction B3 CI not including 1) and the wide CI range overall.

The accurate determination of CP fractions depends, among others, on accurate CP determination. For some CP fractions, differences between calculated and observed values were higher than analytical tolerances for CP analysis (VDLUFA, 2019). This was the case for the A and B1 fraction of compound feeds 1 and 2, and the B3 fraction of compound feed 2. However, for all other CP fractions and compound feeds, the difference between the calculated and observed values of CP fractions is similar or even lower than the analytical tolerance of CP analysis. In addition, small variability between compound feeds (particularly for CP fraction C) probably lowered the accuracy of regression analysis. Consequently, interpretation of additivity for CP fractions is difficult from the results of the present study and different depending on the specific fraction and feed type. Additivity of ED_CNCPS_ was given for all compound feeds. However, the accuracy of regression analysis may be limited owing to the relatively small sample size (*n* = 8 compound feeds) of the present study. Therefore, we recommend to examine the additivity of CP fractions of single feeds in compound feeds in further experiments.

### Effects of pelleting on ruminal fermentation characteristics and feeding value of compound feeds

The second hypothesis of the present study was that the pelleting process would significantly affect GP, dOM, ME, uCP, ID_RUP_ and CP fractions of compound feeds. Based on the present results, this hypothesis can be rejected. Even though the results of statistical analysis indicated an effect of pelleting on GP and related values of ME and dOM, uCP and ID_RUP_, the overall numerical differences were negligible.

When heat is excessively applied during the processing of compound feeds, the intestinal digestibility of protein can be reduced owing to the formation of Maillard products which can neither be fermented nor digested (Sniffen *et al.*, 1992). Any optimum of processing conditions would aim to reduce CP degradability in the rumen without affecting ID_RUP_. The data obtained *in situ* with the same feeds as used in the present study (Grubješić *et al.*, 2019) indicated that pelleting increased rumen degradation of some compound feeds, thus resulting in less RUP entering the small intestine. However, pelleting increased the share of smaller feed particles compared with the mash feeds, which might have increased the number of feed particles leaving the bags without microbial degradation, and thus overestimated degradation. This conclusion is consistent with the results of the present study. In the present study, pelleting increased uCP (which consists of RUP and microbial CP) of most compound feeds (16%, 18%, 20%, 22%, 24% and 26% of CP in DM) up to 24 g/kg DM. No difference was found in the two compound feeds with the highest CP concentrations (28% and 30% of CP in DM).

In a study using duodenally cannulated animals, Goelema *et al.* (1998) did not find an effect on intestinal protein digestibility of mixtures of lupins, peas and faba beans after toasting for 3 min at 132°C. This temperature was higher than the one applied in the present study (pelleting exit temperature of up to 80°C to 90°C). The process of toasting is however technologically not equal to pelleting, as factors other than heat (pressure and moisture) also differ and might result in chemical or physical changes of the substrate. In the present study, except for compound feed 1, ID_RUP_ decreased from 6 to 15 pp in all compound feeds by pelleting.

*In situ* incubations over 16 h were used to generate RUP for *in vitro* determination of ID_RUP_, and results showed that degradation after 16 h increased between 1.4 and 6.4 pp in pelleted compound feeds compared to their corresponding mash feeds. It can therefore be assumed that RUP of mash feeds after *in situ* incubation contained more potentially digestible CP for the *in vitro* enzymatic steps to determine ID_RUP_. This is underlined by the calculation of total tract digestibility (**TTD**) from the summation of 16 h *in situ* RUP and *in vitro* ID_RUP_ which showed that differences in TTD between mash and pelleted compound feeds ranged only between 0.2 and 2.2 pp and can therefore be considered to be negligible. The higher rumen-degraded protein of pelleted compound feeds might be attributed to a smaller particle size compared to mash feeds, as explained in the previous sections.

Pelleting did not have a large effect on CP fractions and ED_CNCPS_ values of compound feeds. Heat treatment during the pelleting process can denaturise protein fraction B2 making it insoluble, resulting in increased B2 and C fractions (Licitra *et al.*, 1996). Such an effect was not found in the present study, probably due to the temperature during pelleting not being very high.

### Prediction of *in situ* ruminal CP degradation from CP fractions

The third hypothesis of the present study was that ED_IN_SITU_ could be predicted using CP fractions. Based on the present results, this hypothesis is rejected. Compared with the corresponding ED_IN_SITU_ data (Grubješić *et al.*, 2019), neither the calculation of ED_CNCPS_ using individual CP fractions and tabular values for their specific degradation rates (Fox *et al.*, 2003) nor ED_CNCPS_ using proximate nutrients and CP fractions based on regression analysis (Shannak *et al.*, 2000) showed adequate prediction accuracy for all compound feeds. However, for two (calculated according to Fox *et al.* (2003)) and three (calculated according to Shannak *et al.* (2000)) out of eight compound feeds, ED prediction with both methods was similar (differences ≤ 3 pp). Attempts of using CP fractions together with proximate nutrients to estimate *in situ* ruminal CP degradation of single and compound feeds showed varying success. Titze *et al.* (2018) reported an overestimation of ED_CNCPS_ of lupins using the approach of Fox *et al.* (2003), for an average of 10 pp. In the present study, ED_CNCPS_ was generally lower than ED_IN_SITU_ for all compound feeds and prediction accuracy was very variable with differences from 1 to 14 pp. A problem when using the approach of Fox *et al.* (2003) is the necessity of using tabulated values for the degradation rate of the specific CP fractions. It was not mentioned how degradation rates were obtained, how many samples the provided mean values are based on and how high the range of degradation rates for individual CP fractions of the same feedstuff was. Shannak *et al.* (2000) derived their prediction equations from selected proximate nutrients and CP fractions for *in situ* RUP values including 11 dairy compound feeds. Therefore, prediction of ED of compound feeds may be possible with good accuracy. Shannak *et al.* (2000) found differences between *in situ* RUP values and respective estimates of up to 79 g/kg CP; however, 8 out of 11 RUP values had differences ≤ 50 g/kg CP. For samples of the present study, ED_IN_SITU_ and ED_CNCPS_ calculated according to Shannak *et al.* (2000) differed by up to 16 pp and hence 5 out of 8 compound feeds had differences between estimated and *in situ* RUP ≥ 100 g/kg CP for *k* = 5 and 8%/h. Poor estimation may result from differences in the assay details because NDF was determined by manual filtration in the study of Shannak *et al.* (2000), and authors stated that results may deviate from those obtained with the conventional NDF method which was used in the present study. Moreover, NDF values ranged between 212 and 554 g/kg DM in the 11 compound feeds of Shannak *et al.* (2000) and only between 142 and 255 g/kg DM in the present study. Shannak *et al.* (2000) also included forages and special by-products in the development of the regression equations, which is another difference to the present study. It is therefore recommended to extend the existing database. More accurate equations may be developed when covering a wider range of feedstuff groups.

## Conclusion

We conclude that, when formulating compound feeds for cattle, single feed data for GP_24_, dOM, ME and uCP are additive, while those for ID_RUP_ are not. Additivity of CP fractions is dependent on the fraction and compound feed type, whereas ED_CNCPS_ is precisely additive. The pelleting process had little effect on ruminal fermentation characteristics and feeding values of compound feeds, probably because heat exposure was moderate. Using CP fractions in the present study did not reliably predict *in situ* ruminal CP degradation of compound feeds: more studies are needed to extend the database for the development of prediction equations.

## Software and data repository resources

No data were deposited in an official repository.
